# Supplementary material for: Aerosol breezes drive cloud and precipitation increases
Source: Nat Commun. 2023 May 2;14:2508. doi: 10.1038/s41467-023-37722-3 (PMC10154388; doi:10.1038/s41467-023-37722-3)
Supplement: Supplementary file 1 — Supplementary Information [file 41467_2023_37722_MOESM1_ESM.pdf]

Supplementary Information for

Aerosol breezes drive cloud and precipitation increases

Gabrielle R. Leung[1] and Susan C. van den Heever[1]

[1] Department of Atmospheric Science, Colorado State University, Fort Collins, CO

Corresponding author: Gabrielle R. Leung (gabrielle.leung@colostate.edu)

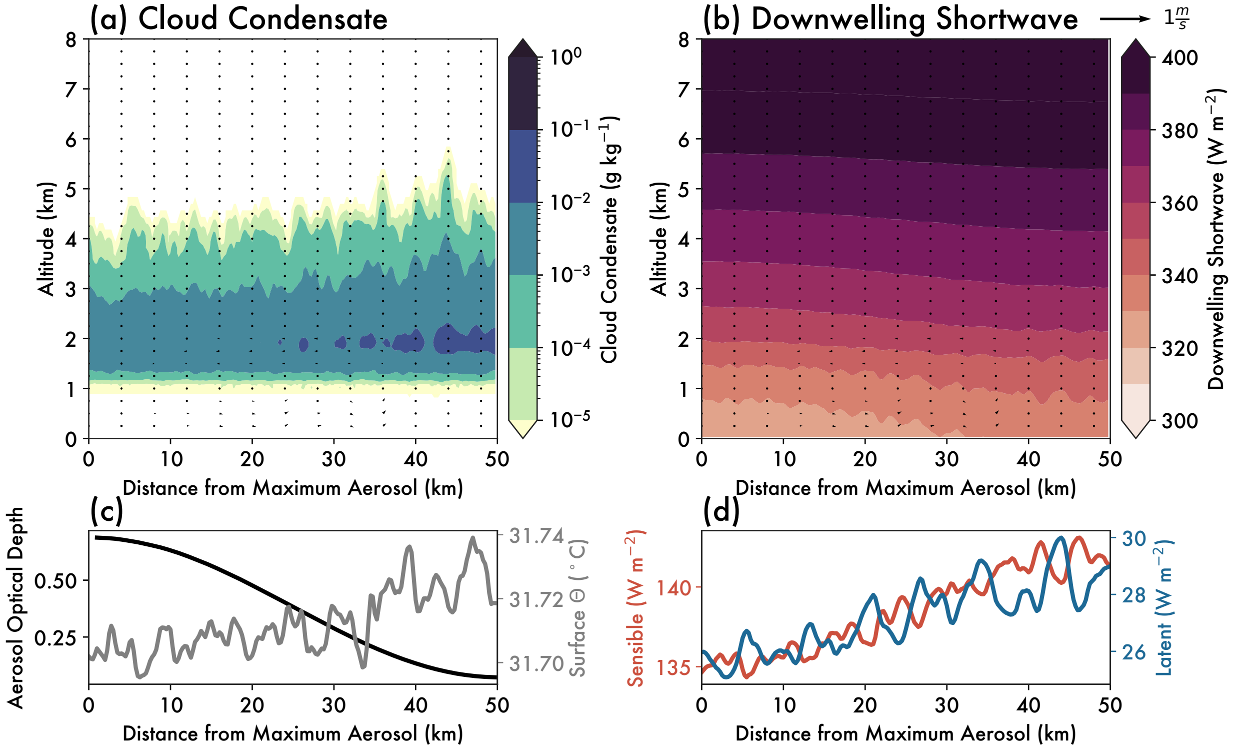

Supplementary Figure 1. Aerosol breeze circulation from scattering aerosol reduced-gradient simulation. Mean cross section through the domain over all 12 hours of the scattering aerosol reduced gradient simulation, averaged temporally and zonally. The abscissa is given as a function of distance from the domain center/maximum aerosol concentration (horizontal black line in Figure 6b,c), such that the high-aerosol region is on the left and the low-aerosol region is on the right of these panels. Shading in (a) shows cloud condensate mixing ratios (g kg^{-1}), and in (b) the downwelling shortwave flux (W m^{-2}). The wind barbs in (a) and (b) show the mean vertical and horizontal winds oriented along the aerosol gradient. (c) depicts the aerosol optical depth at the surface in black (left y-axis) and the surface potential temperature in gray (right y-axis; ºC), while (d) depicts the sensible surface heat flux in red (left y-axis; W m^{-2}) and latent surface heat flux in blue (right y-axis; W m^{-2}), all averaged temporally and zonally.

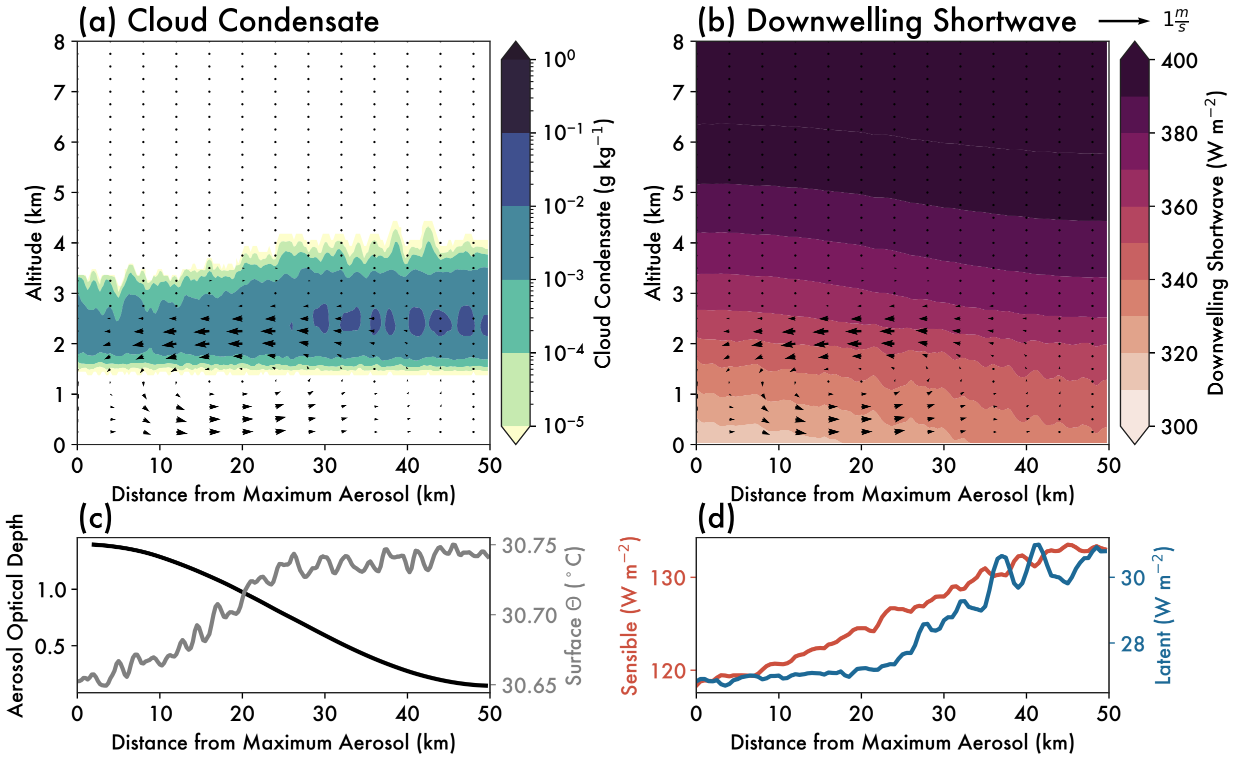

Supplementary Figure 2. Aerosol breeze circulation from scattering aerosol gradient simulation using midlatitude sounding. Mean cross section through the domain over all 12 hours of the midlatitude scattering aerosol gradient simulation, averaged temporally and zonally. The abscissa is given as a function of distance from the domain center/maximum aerosol concentration (horizontal black line in Figure 6b,c), such that the high-aerosol region is on the left and the low-aerosol region is on the right of these panels. Shading in (a) shows cloud condensate mixing ratios (g kg^{-1}), and in (b) the downwelling shortwave flux (W m^{-2}). The wind barbs in (a) and (b) show the mean vertical and horizontal winds oriented along the aerosol gradient. (c) depicts the aerosol optical depth at the surface in black (left y-axis) and the surface potential temperature in gray (right y-axis; ºC), while (d) depicts the sensible surface heat flux in red (left y-axis; W m^{-2}) and latent surface heat flux in blue (right y-axis; W m^{-2}), all averaged temporally and zonally.

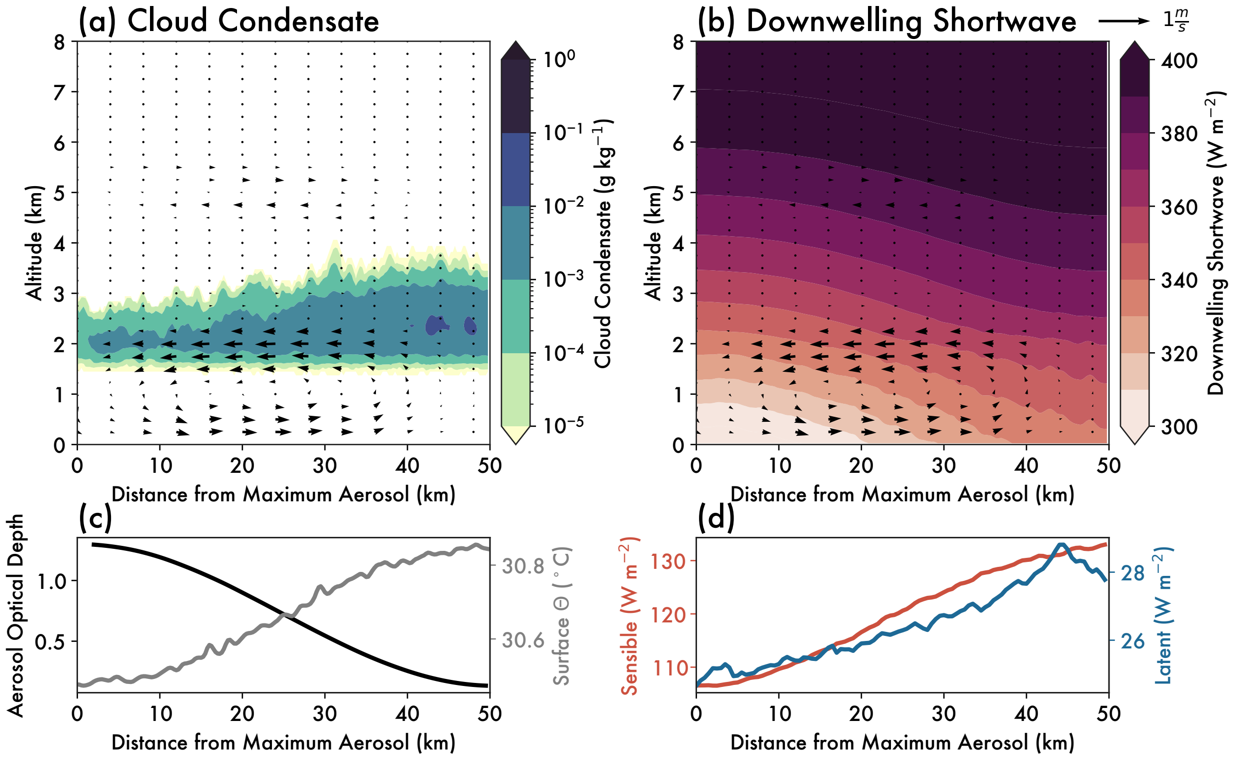

Supplementary Figure 3. Aerosol breeze circulation from absorbing aerosol gradient simulation using midlatitude sounding. Mean cross section through the domain over all 12 hours of the midlatitude absorbing aerosol gradient simulation, averaged temporally and zonally. The abscissa is given as a function of distance from the domain center/maximum aerosol concentration (horizontal black line in Figure 6b,c), such that the high-aerosol region is on the left and the low-aerosol region is on the right of these panels. Shading in (a) shows cloud condensate mixing ratios (g kg^{-1}), and in (b) the downwelling shortwave flux (W m^{-2}). The wind barbs in (a) and (b) show the mean vertical and horizontal winds oriented along the aerosol gradient. (c) depicts the aerosol optical depth at the surface in black (left y-axis) and the surface potential temperature in gray (right y-axis; ºC), while (d) depicts the sensible surface heat flux in red (left y-axis; W m^{-2}) and latent surface heat flux in blue (right y-axis; W m^{-2}), all averaged temporally and zonally.

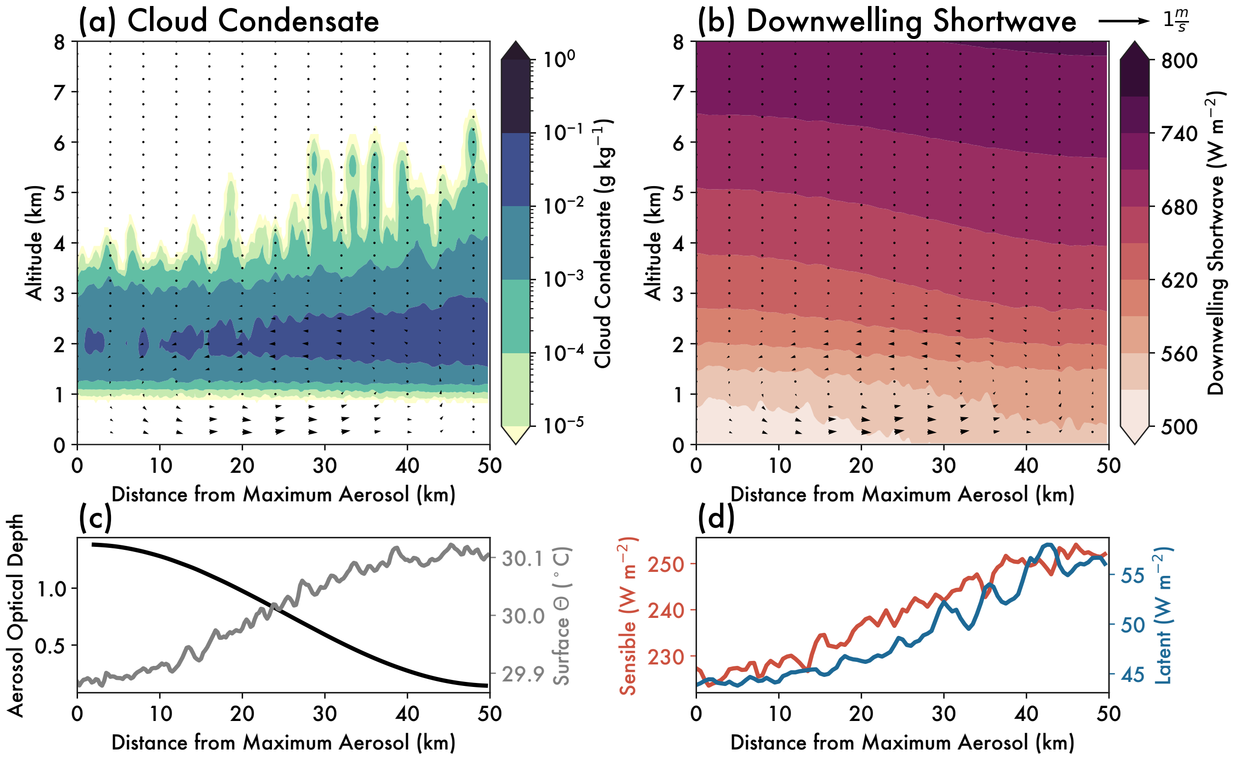

Supplementary Figure 4. Aerosol breeze circulation from scattering aerosol gradient simulation with diurnal cycle on. Mean cross section through the domain between 11AM-12NN (approximately four hours after sunrise) for the scattering aerosol gradient simulation with the diurnal cycle fully represented, averaged temporally and zonally. The abscissa is given as a function of distance from the domain center/maximum aerosol concentration (horizontal black line in Figure 6b,c), such that the high-aerosol region is on the left and the low-aerosol region is on the right of these panels. Shading in (a) shows cloud condensate mixing ratios (g kg^{-1}), and in (b) the downwelling shortwave flux (W m^{-2}). The wind barbs in (a) and (b) show the mean vertical and horizontal winds oriented along the aerosol gradient. (c) depicts the aerosol optical depth at the surface in black (left y-axis) and the surface potential temperature in gray (right y-axis; ºC), while (d) depicts the sensible surface heat flux in red (left y-axis; W m^{-2}) and latent surface heat flux in blue (right y-axis; W m^{-2}), all averaged temporally and zonally.
